# Dietary Health Behaviors of Women Living in High Rise Dwellings: A Case Study of an Urban Community in Malaysia

**DOI:** 10.1007/s10900-012-9597-1

**Published:** 2012-08-29

**Authors:** Tilakavati Karupaiah, Winnie Chee Siew Swee, Siew Ying Liew, Boon Koon Ng, Karuthan Chinna

**Affiliations:** 1 Faculty of Health Sciences, School of Healthcare Sciences, National University of Malaysia, Jalan Raja Muda Abdul Aziz, 50300 Kuala Lumpur, Malaysia; 2 Department of Nutrition and Dietetics, International Medical University, Bukit Jalil, 57000 Kuala Lumpur, Malaysia; 3 Department of Social and Preventive Medicine, University of Malaya, Lembah Pantai, 56750 Kuala Lumpur, Malaysia

**Keywords:** Urban women, High rise dwelling, Ethnicity, Income, Diet quality, HEI

## Abstract

Diet-related non-communicable disease (DR-NCD) occurrence is a serious problem amongst Malaysian women and urbanization is probably a challenge to their achieving the nutritional environment conducive to healthy eating. This case study aimed to determine diet quality of an urban community using women respondents from high rise dwellings in Kuala Lumpur. The sample consisted of 135 households and a healthy eating index (HEI) scale was used to evaluate the women’s diet quality. A total of 128 women (Malays = 45, Chinese = 56, Indian = 27) participated. Total HEI score was significantly different (*P* < 0.05) within ethnicity (Indians = 75.7 ± 8.1 <Malays = 80.5 ± 7.4 <Chinese = 80.1 ± 8.1) and affected by component scores for fruit (range 3.8–6.2, *P* = 0.044), sodium (range 7.8–9.0, *P* = 0.006) and food variety (range 9.3–9.9, *P* = 0.001). Dairy foods rated poorly (range 2.0–3.9, *P* > 0.05) regardless of ethnicity. Income strata (ρ = 0.159, *P* = 0.048) and eating out frequency (ρ = −0.149, *P* = 0.046) also independently affected HEI scores. Income negatively correlated with sodium restriction score (ρ = −0.294, *P* = 0.001) but positively with cereals (ρ = 0.181; *P* = 0.025), fruits (ρ = 0.178; *P* = 0.022), dairy products (ρ = 0.198; *P* = 0.013) and food variety (ρ = 0.219, *P* = 0.007). Decreased vegetable intake (ρ = −0.320; *P* < 0.001) and sodium excess (ρ = −0.135, *P* = 0.065) were associated with eating out frequency and poor HEI scores. This case study suggests health promotion for DR-NCD prevention is needed at the community level to improve diet quality of urban women.

## Introduction

In tandem with rising urbanization and economic growth in Malaysia over 3 decades, incidence of diet-related non-communicable diseases (DR-NCD) such diabetes, hypertension and cardiovascular diseases has been increasing exponentially [1, 2]. Women’s health in Malaysia is largely affected by DR-NCDs. In 2008 of 28,936 certified deaths amongst Malaysian women, 10.6 % was from ischemia, 7.7 % from CVD and 3.0 % from malignant neoplasm of breast [3]. Of note, 12,070 of these deaths belonged to women in the 15–64 years age group and attributable to 8.3 % for ischemia, 6.7 % from CVD and 6.0 % for breast cancer.

Globally, public health intervention efforts to curb the occurrence of DR-NCDs have been to introduce food-based dietary guidelines (FBDG) with a commonality of emphasis on whole grains, fruits and vegetables [4–7]. The nationwide Malaysian Adult Nutrition Study (MANS) published population nutrient intakes for energy, protein, fats and other macronutrients although the ability of specific groups to consume healthful foods was not reported [8]. In contrast the overall average monthly household expenditure in Malaysia rose by 12.1 % to RM 2190 in 2009/2010 compared to 1993/1994 with 20.3 % of the household budget spent on just food excluding alcohol and cigarettes [9]. Urban households alone spend 25 % of their budget on food. It was noted that for these contrasting years the proportion of the household budget fell for fruit (2.2–1.2 %) and vegetable (2.9–2.1 %) purchases with other food groups remaining unaffected.

The informational gap in public health monitoring perhaps highlights a need for community level health monitors to use simple indicators to measure diet quality and understand factors affecting food choice. In most societies, women are the major decision makers about food provision for their families [10, 11]. This role of women is expectedly critical in view of the rise in diet-related non-communicable diseases (DR-NCD) such diabetes, hypertension and cardiovascular diseases affecting adulthood irrespective of gender. These women, as heads of their households, would also be responsible for contributing to the home nutritional environment and influence lifestyle practices in their children.

We hypothesize that the ability of urban communities to meet healthy eating guidelines will be affected by food accessibility in a built environment and the matrix of factors affecting food choices would be unique to their built environment. Adherence to nutritious food choices was measured through the assessment of diet quality using the Healthy Eating Index (HEI) [12]. Understanding factors affecting women’s HEI would help to identify barriers to healthful food choices. Diet quality was defined by the key messages of the Malaysian Food Pyramid (MFP) in terms of serving size [13].

## Methodology

### Study Design

This was a case study about the diet quality of an urban community of women living in high rise dwellings and having access to similar food retail services. Diet quality was assessed using a HEI instrument developed by reference to the Malaysian Food Pyramid (Table 1) [13]. This study received ethical approval from the Institutional Review Board of the National University of Malaysia.

**Table 1:** Dietary determinants for HEI scoring

Components^a^	Range of score	Scoring criteria	
		Maximum (=score of 10)	Minimum (=score of zero)
*Grouping A: nutritional adequacy*			
Cereals	0–10	8–12 servings	0 servings consumed
Vegetables	0–10	3 servings	0 servings consumed
Fruits	0–10	2 servings	0 servings consumed
Dairy products	0–10	1–2 servings	0 servings consumed
Protein foods^b^	0–10	2–3 servings	0 servings consumed
*Grouping B: moderated intake*			
Total fat	0–10	≤30 % total energy intake	≥45 % total energy intake
Saturated fat	0–10	≤7 % total energy intake	≥15 % total energy intake
Cholesterol	0–10	≤300 mg	≥450 mg
Sodium	0–10	≤2,400 mg	≥4,800 mg
*Grouping C: optimized choice*			
Food variety	0–10	Inclusion of ≥ 16 food varieties over 3 days	≤ 6 food varieties over 3 days

Adapted from Kennedy et al [12]

^a^Based on Malaysian food pyramid [13]

^b^Meat/poultry/ seafood/ legumes

### Study Location, Sampling and Description of Community

The study was conducted in Kuala Lumpur, the capital city of Malaysia. The primary index in 2000 for major metropolitan towns in Malaysia ranked Kuala Lumpur as the largest urbanized city with a population of 1,305,800 and several clusters of high rise medium and low cost high rise dwellings close to the urban city center [14]. In this study, one of these clusters was selected randomly. Then, in this cluster two blocks were selected randomly. Within these two blocks, 135 households were selected based on voluntary basis.

The randomly chosen cluster of high rise dwellings was within 3 km of the metropolitan area of Kuala Lumpur. The communities in the selected cluster had access to 3 major supermarkets, 4 convenience stores and 4 wet markets selling fresh produce. Neighborhoods were multiethnically diverse with Malay, Chinese and Indian households. However, according to the 2009 census, a total of 6,409 households were registered with the Local Authority Council but 20 % of these households were occupied by male foreign migrant workers [15].

### Subject Selection

Recruitment of women from households in the 2 blocks within the cluster came through the distribution of leaflets. Exclusion criteria were households comprising migrant foreign workers, bachelors, student groups or pregnant or lactating women or women with disabilities that would hinder the collection of self-recorded dietary records. Flyers were distributed at food retail centers in the neighborhood and in all women from 135 households consented to participate in the study.

### Study Period

The study was conducted between September 2008 and February 2009, and participation during this period was adjusted to ensure participants were not celebrating an event according to their religious/ethnic denomination.

### Subject Demographics

Questionnaires were administered to collect data on socio-demographic status, household income and food consumption patterns of participants including eating out habits. Eating habits were based on the definition of Lin et al [16] by which ‘eating at home’ included foods prepared at home from raw ingredients whereas ‘eating out’ included all foods that were purchased in a ready-to-eat state and identified as foods purchased from hawkers, night markets, restaurants, canteens or retail shops or convenient foods such as frozen foods [16]. The questionnaire content was developed based on the *Malaysian Household Expenditure Survey (HES*) and pre-tested for validity on 25 women at a different location [17].

### Anthropometry

The body mass index (BMI) of participants was calculated from the formula, weight (kg) divided by height (m^2^)

### Assessment of Food Consumption Patterns

Dietary habits were evaluated from 3 days’ dietary records (3DDR) submitted by subjects representing two week-days and one week-end intakes . To ensure accuracy of information participants were pre-trained in recording portion size of the different food groups using standard household measuring units and pictures of food serving sizes [18]. The prerequisite was the uniformity in recording consumed food groups according to standardized household measures which were converted into portion size by weight [19]. The nutritional values of consumed foods were analyzed based on appropriate food databases as well as food labels of pre-packaged foods [20–23]. Submitted 3DDRs of participants were tested for under- and over-reporting [24]. On this basis five undereporters (EI/BMR < 1.2) and two overeporters (EI/BMR > 1.8) were excluded from the sampling frame in order to control for outliers [25]. Finally 3DDR data from 128 participants were used in the HEI analysis.

### Assessment of Diet Quality

The methodology was designed to estimate the food consumption patterns of different food groups based on a comparative guide to healthy eating as described by the original HEI [12]. Accordingly, food consumption was tested covering a total of 10 components (1) Grouping A included basic food groups comprising cereals, vegetables, fruits, dairy products and protein foods (2) Grouping B included four components based on total fat, saturated fat, cholesterol and sodium and (3) Grouping C with one component testing food variety. Each component was tested on a scale of one to 10 using the adherence to dietary guidelines as a reference basis. Assignment of scores for each component was based on proportional valuation. For example, cereal consumption of eight or more servings were assigned a score of 10, whereas consuming four servings received a score of five or consuming six servings received a score of 7.5.

Totaled HEI individual scores were collectively categorized into <51 (poor), 51–80 (needs improvement) and >80 (good). Table 1 explains these 10 components and the scoring criteria based on the MFP [13].

### Data Interpretation

The consumption of each food component listed in HEI was quantified from 3DDR data by conversion of absolute weight into portion size [20]. Components within Grouping A (encouraged intake) were quantified into serving sizes as recommended by the MFP [13]. For the four components within Grouping B (moderated intake) values were based on nutrient analysis as per the food databases. Food variety in Grouping C was based on counting the number of different types of foods extracted from 3DDR data.

Data on nutrient intake for each participant was averaged from 3DDR and consolidated as per age group specified by the Recommended Nutrient Intake (RNI) guidelines for Malaysians [26].

### Statistical Analyses

Mean ± SD and percentages were used for descriptive data on sociodemographic details as well as reporting nutrient intake data and HEI scores. Comparisons in total HEI scores among categories of age, ethnicity, BMI, education or employment status were performed with ANOVA testing. Significance in individual HEI component scores was detected by Kruskal-Wallis testing. Post-hoc validation using Spearman rank correlation coeffcient analysis assessed associations between total HEI score and component scores with non-normally distributed data. Data analysis was carried out with the *Statistical Package for Social Sciences* (SPSS) version 15.0 software. Significance was interpreted at *P* < 0.05.

## Results

Demographic data of participating women (n = 128) including age, ethnicity, BMI, religion, educational status, employment and monthly income is presented in Table 2. Most of the interviewed women were in the young to middle-aged years (<29 years, n = 19; 30–50 years, n = 85; 51–59 years, n = 19) with very few elderly women (>60 years, n = 5).

**Table 2:** Sociodemographic characteristics of study subjects

Characteristics	Subjects (n = 128)	
	‘n’	%
Age (years)		
21–30	19	14.8
31–50	85	66.4
51–59	19	14.8
>60	5	3.9
Ethnicity		
Malays	45	35.2
Chinese	56	43.7
Indians	27	21.1
Religion		
Muslim	45	35.2
Buddhist	50	39.0
Hindu	26	20.3
Christians	7	5.5
Educational status		
Primary	21	16.4
Mid-High school	24	18.8
Completed high school	55	43.5
Diploma/ degree	27	21.1
Employment		
Government institutions	10	7.8
Private institutions	35	27.3
Self-employed	12	9.4
Housewife	71	55.5
Monthly income levels		
<RM 1500	31	24.2
RM 1500–RM 3500	63	49.2
RM 3500–RM 5500	20	15.6
>RM 5500	14	11.0

1 US Dollar = RM 3.15

### Nutritional Intake as per RNI

Data on nutrient intake of participants obtained from 3DDR is presented in Table 3 as per age group. Nutritional adequacy interpreted as meeting RNI was sufficient for most nutrients except calcium. Intake of calcium below RNI was evident for all age groups. Additionally 27 % of women <30 years did not meet RNI for thiamine whilst 48 % of both women groups <29 years and 30–50 years did not meet RNI for iron needs.

**Table 3:** Nutrient intake data of subjects by age categories (n = 128)

Nutrient	19–29 years (n = 19)			30–50 years (n = 85)			51–59 years (n = 19)			60–65 years (n = 5)		
	Mean ± SD	RNI	% RNI	Mean ± SD	RNI	% RNI	Mean ± SD	RNI	% RNI	Mean ± SD	RNI	% RNI
Energy (kcal)	1,867 ± 175	2,000	93	1,838 ± 229	2,180	84	1,706 ± 109	2,180	78	1,703 ± 55	1,780	96
Carbohydrate (g)	260 ± 37	–	–	260 ± 43	–	–	248 ± 40	–	–	241 ± 15	–	–
Protein (g)	71 ± 9	55	129	71 ± 11	55	129	71 ± 11	55	129	60 ± 11	51	118
Fat (g)	53 ± 4	–	–	53 ± 7	–	–	50 ± 5	–	–	49 ± 5	–	–
Vitamin A (μg)	832 ± 247	500	166	732 ± 281	500	146	699 ± 262	500	140	972 ± 248	500	194
Thiamine (mg)	0.8 ± 0.3	1.1	73	1.0 ± 0.7	1.1	92	1.0 ± 0.3	1.1	91	2.1 ± 2.8	1.1	191
Riboflavine (mg)	1.3 ± 0.4	1.1	118	1.2 ± 0.3	1.1	79	1.5 ± 0.5	1.1	136	2.6 ± 3.6	1.1	236
Niacin (mg)	14 ± 2.8	14	100	14 ± 3.4	14	100	15 ± 2.7	14	107	14 ± 1.3	14	100
Vitamin C (mg)	88 ± 45	70	126	100 ± 74	70	143	104 ± 46	70	149	83 ± 65	70	119
Calcium (mg)	489 ± 145	800	61	462 ± 138	800	58	511 ± 137	1,000	51	474 ± 147	1,000	47
Iron (mg)	15 ± 4	29	52	15 ± 4	29	52	17 ± 5	11	155	15 ± 5	11	136
Sodium (mg)	2,833 ± 431	–	–	2,724 ± 517	–	–	2,709 ± 349	–	–	2,683 ± 435	–	–
Dietary fibre (g)	17.36 ± 8.40	–	–	20.07 ± 8.08	–	–	21.35 ± 10.11	–	–	21.01 ± 6.77	–	–

By comparison with the Malaysian recommended nutrient intake (RNI) for adults women where applicable [26]

### Diet Quality

The overall diet quality assessed by the HEI score (mean ± SD) was 79.3 ± 8.0 and classified as ‘need improvement’ (Table 4). About 55.5 % of women (n = 71) were identified in this category (HEI score = 73.6 ± 5.7) whilst the remaining 44.5 % (n = 57) achieved ’good’ diet quality (HEI score = 86.4 ± 3.4). Consumption of most Grouping A food groups met recommended MFP serving size for nutritional adequacy except for dairy products (HEI score = 3.1 ± 3.8) <fruits (HEI score = 5.6 ± 4.1) <vegetables (HEI score = 7.4 ± 3.0). Reported mean serving size for consumption of dairy foods was 0.3, which was much below the recommended 1–2 servings of the MFP. For components in Grouping B, mean HEI scores were close to the maximum of 10 indicating the ability to keep within the recommended restriction for total fat, saturated fat, cholesterol and sodium. Grouping C which tested variety in food selection to promote a balanced diet, subjects did not achieve the maximum score of 10 (HEI score = 9.7 ± 0.7) because only 13 types of food items were consumed over 3 days instead of the targeted 16 different items.

**Table 4:** Total and component HEI scores by ethnic groups

HEI categories	Mean servings achieved day^−1^	Total	Malay	Chinese	Indian	*P* value
		(n = 128)	(n = 45)	(n = 56)	(n = 27)	
Total HEI score		79.3 ± 8.0	80.5 ± 7.4	80.1 ± 8.1	75.7 ± 8.1	0.026^a,^*
HEI rating						
Poor		–				
Improvement required		73.6 ± 5.7				
Good quality		86.4 ± 3.4				
Grouping A						
Cereals	7	8.5 ± 1.3	8.6 ± 1.3	8.3 ± 1.3	8.7 ± 1.2	0.144^b^
Vegetables	2	7.4 ± 3.0	7.0 ± 3.3	8.0 ± 2.7	7.1 ± 2.8	0.164^b^
Fruits	1	5.6 ± 4.1	6.2 ± 3.9	6.0 ± 4.0	3.8 ± 4.0	0.044^b,^*
Dairy products	0.3	3.1 ± 3.8	3.9 ± 4.0	2.9 ± 3.6	2.0 ± 3.3	0.096^b^
Meat & legumes	2	9.4 ± 1.3	9.6 ± 0.8	9.5 ± 1.1	8.7 ± 2.1	0.057^b^
Grouping B						
Total fat	26 % en	10.0 ± 0.1	9.9 ± 0.3	10.0 ± 0.1	10.0 ± 0.1	0.455^b^
Saturated fat	8 % en	8.2 ± 2.0	8.2 ± 1.9	8.5 ± 1.9	7.7 ± 2.3	0.143^b^
Cholesterol	237 mg	9.1 ± 2.1	8.7 ± 2.7	9.3 ± 1.7	9.4 ± 1.4	0.723^b^
Sodium	2,737 mg	8.4 ± 1.7	8.6 ± 1.5	7.8 ± 1.9	9.0 ± 1.0	0.006^b,^*
Grouping C						
Food variety	13 types/ 3 days	9.7 ± 0.7	9.6 ± 0.8	9.9 ± 0.3	9.3 ± 1.1	0.001^b,^*

Scores are reported as mean ± SD

* Significance at *P* < 0.05

^a^ *ANOVA* tested significant for overall total HEI scores by racial grouping

^b^ *Kruskal*-*Wallis* testing for component HEI scores by racial grouping

### Factors Influencing HEI

#### Ethnicity

**Fig. 1:**
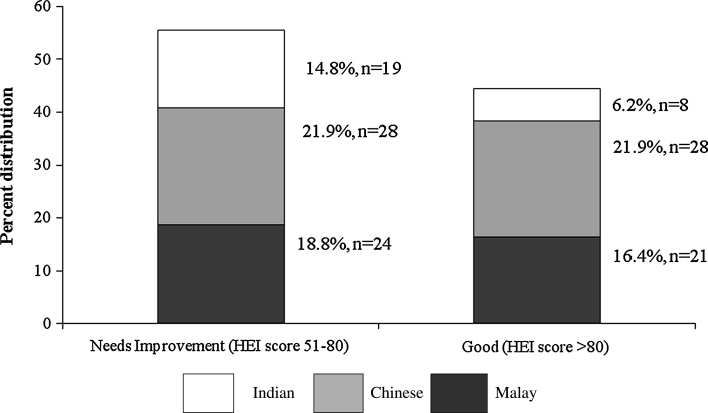
Distribution of racial groups as per HEI rating to determine diet quality. Distribution by ethnicity as per HEI rating indicates ~70 % of Indian diets were categorised as ‘need improvement’ compared to 50 % of Chinese and 53 % of Malays. *HEI* healthy eating index

Ethnicity of the women independently affected HEI scores (*P* = 0.026). Figure 1 gives the distribution of ethnicity as per the HEI rating for diet quality indicating ~70 % of Indians fell into the category of ’need improvement’ compared to 50 % of Chinese and 53 % of Malays. Indian diets scored the lowest HEI score of 75.7 ± 8.1 (categorized as ’need improvement’) compared to Malay (HEI score = 80.5 ± 7.4) and Chinese (HEI score = 80.1 ± 8.1) scores (Table 4). The components that affected HEI score for Indians were fruit (HEI score = 3.8 ± 4.0, *P* = 0.044) and dairy product (HEI score = 2.0 ± 3.3, *P* = 0.096) consumption. Sodium intake was significantly affected also by ethnicity (*P* = 0.006) and in the order of Indians (HEI score = 9.0 ± 1.0) <Malays (HEI score = 8.6 ± 1.5) <Chinese (HEI score = 7.8 ± 1.9). The ethnic factor was also significantly affected (*P* = 0.001) food variety optimization with Indians least able to achieve food variety in the diet compared to the other ethnic groups.

#### Monthly Income

Apart from ethnicity, better total HEI scores were associated with larger monthly incomes (ρ = 0.159, *P* = 0.048) (Table 5). Income size was positively correlated to achieving nutritional adequacy for cereals (ρ = 0.181, *P* = 0.025), fruits (ρ = 0.178, *P* = 0.022), dairy products (ρ = 0.198, *P* = 0.013) and food variety (ρ = 0.219, *P* = 0.007) but negatively with sodium restriction (ρ = −0.294, *P* = 0.001).

**Table 5:** Other factors correlating with HEI scores

HEI components	Monthly income		Eating out	
	Spearman rho (ρ)	*P* value	Spearman rho (ρ)	*P* value
Total HEI score	0.159	0.048*	−0.149	0.046*
Grouping A				
Cereals	0.181	0.025*	0.074	0.203
Vegetables	0.060	0.252	−0.320	0.000*
Fruits	0.178	0.022*	−0.004	0.483
Dairy products	0.198	0.013*	0.071	0.214
Meat and legumes	0.089	0.159	0.037	0.339
Grouping B				
Total fat	0.056	0.263	−0.023	0.397
Saturated fat	−0.060	0.207	−0.072	0.209
Cholesterol	0.030	0.370	−0.043	0.314
Sodium	−0.294	0.001*	−0.135	0.065
Grouping C				
Food variety	0.219	0.007*	0.006	0.472

Analysis between factors by Spearman rank correlation coefficients to test non-nominally distributed data. * Statistical significance for *P* < 0.05

**Fig. 2:**
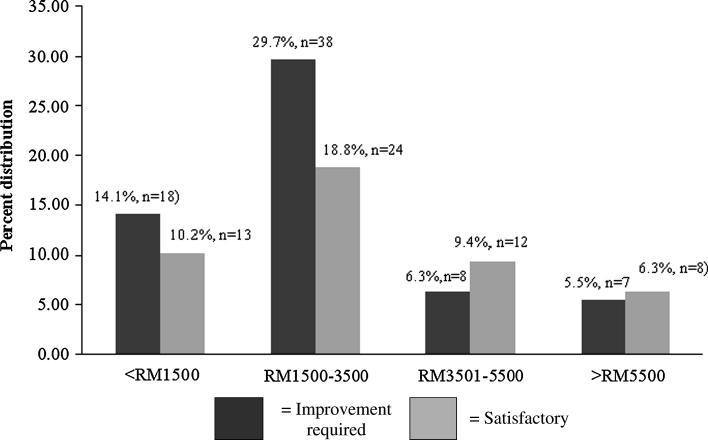
HEI scoring for diet quality as per income strata. Indicates income strata and diet quality categories. Subjects not achieving satisfactory HEI scores (<80) were more likely to be in the lower income brackets (<RM 1500 = 14.06 % and RM 1500–3500 = 29.69 %) compared to those with satisfactory scores (>80) with incomes >RM 3500. *HEI* healthy eating index

Income strata and diet quality categories are indicated in Fig. 2. Percent participants not achieving satisfactory HEI scores (<80) were greater in the lower income brackets (<RM 1500 = 14.06 % and RM 1500–3500 = 29.69 %) whereas satisfactory scores (>80) were more likely with incomes >RM 3500. The trend of satisfactory HEI scorers (>80) was-41.9 % (<RM 1500), 40. % (RM 1500–RM 3500), 60 % (RM 3501–RM 5500) and 60 % (>RM 5500).

#### Eating out Frequency

Diet quality for participants consuming home prepared meals was ‘good’ (17.2 %, 82.0 ± 8.0) compared to those eating one to two meals outside (68.8 %, 78.8 ± 7.8) or all meals outside (14.0 %, 78.3 ± 8.9). Consuming meals outside was negatively correlated with HEI score (ρ = −0.149, *P* = 0.046) with a decline in vegetable intake being a major factor (ρ = −0.320, *P* < 0.001). A similar trend in sodium intake was observed but was not significant (ρ = −0.135, *P* = 0.065) (Table 5).

#### Other Factors

BMI, age, educational and employment status of the women did not affect HEI scores (*P* > 0.05). However, in categorizing BMI range, the diet quality of women with BMI < 18.5 was related to a poorer HEI score (71.7, *P* = 0.016) compared to the higher categories (>77.9, *P* > 0.05).

## Discussion

The federal territory of Kuala Lumpur has the highest mean monthly household income of RM 4930 with the highest urbanization level of 100 % in Malaysia [27]. This depicts the characteristic of a developing society whereby employment in the agricultural sector diminishes and in-line with Reissman’s definition that urbanization is the transformation of rural agricultural societies into industrial urban ones (1970) [28]. However social development has not been on par with the rapidly growing economy and the extensive physical development that has taken place in Kuala Lumpur [27]. By ranking for social development indicators within Malaysia, Kuala Lumpur which is Malaysia’s capital city is ranked no. 9 (average score of 7.00, range 3.88–9.50) of 14 states inclusive of federal territories. Schooling and Leung (2010) believe the current evidence from the developed world may be largely uninformative for preventing or mitigating social disparities in non-communicable chronic diseases in developing countries [29]. They suggest rapid economic transition in the developing world provides a unique opportunity to re-examine the origins of, and biological mechanisms driving social disparities and research efforts should focus on material resources, civic infrastructure and social structure of societies. Evaluation of the nutritional environment is the first step in creating a healthy eating environment. To fit in with this spirit of enquiry for the prevention of DR-NCDs, this case study focuses on the question whether women’s habitual food patterns in an urban environment reflect desirable nutritional behaviors as recommended by food-based dietary guidelines (FBDG) and what are the social determinants affecting their food choices. Women are the decision-makers about dietary behavior within a household and therefore not only their health but health of family members within their household will be affected by their food choices.

Along with poor social indicators for Kuala Lumpur and despite a higher household income compared to the rest of Malaysia, the cost of living in an urban location is very much higher (housing, transport, loan repayments) with data indicating that budgeted expenditure for food, such as fruits and vegetables is falling [27]. In this case study, low HEI scores (<51) were not evident in these women in agreement with overall adequate nutritional intake judged by comparison with the Malaysian RNI [26]. However, less desirable HEI scores were affected by choices of specific food groups. Component HEI scores for dairy products, fruits and vegetables indicated the women were not meeting suggested serving sizes recommended by the MFP. In particular the mean serving size of 0.3 for dairy foods was much below the recommended 1–2 servings of the MFP. This is consistent with findings from the Malaysian Adult Study (MANS) which reported mean calcium intake of less than 50 % of the RNI [8].

Our findings identified ethnicity as a key determinant of HEI scores of women in this study. Seventy percent of Indian diets were categorized into ‘needs improvement’ compared to Chinese (50 %) and Malay (53 %) diets. In analysis, diet quality of Indian women was affected by poor HEI scores for fruit, dairy foods and food variety whereas Chinese women scored the lowest for been able to moderate sodium intake. Ethnicity is a determinant in the development of personal factors including culture and ethnicity which affect choice and selection of food [30]. This is particularly true in the choice of meats in Malaysia which is affected by religious practices-Muslims prefer beef whilst Indians who are largely Hindus will only eat mutton, chicken or fish or practice vegetarianism.

Another key determinant of HEI scores was household income of this group of women. Income size was positively correlated to achieving nutritional adequacy for cereals, fruits, dairy products and food variety but negatively with sodium restriction. Meal frequency whether prepared at home or bought outside the home also affected diet quality. Outside meals were negatively correlated with HEI scores with a decline in vegetable intake being a major factor. A trend in decreased adherence to sodium restriction was also observed with outside meals. Some of our findings appear to support the energy-cost argument based on selected cross-sectional studies in England, France and USA [31–33]. Drewnowski’s group have hypothesized that family income may be related to diet quality in part because palatable, energy-dense foods, such as fats and sugars, are inexpensive on an energy-cost basis compared with healthier foods, such as fruits and vegetables [31–33]. We did not assess sugars as our food database does not provide this information. But in agreement with the energy-cost hypothesis, there are positive associations between income and fruit and vegetable consumption. Conversely, our results did not indicate a positive association between ethnic/ income and fat consumption. This is perhaps due to the overall mean fat consumption of 26 % en which is well below the <30  % en recommendation in the MFP [13]. Another association that is solely linked to ethnic/ income but negatively was sodium consumption.

Income disparity between households affected diet quality given the same accessibility to food. This was observed in the case study which looked at an urban community of women living in medium cost high rise dwellings within a single neighbourhood indicated the ability to purchase a quality diet is affected by income. This segment of urban society represents a vulnerable group who are at risk for DR-NCDs. Further this vulnerable group of women in turn are responsible for the diet quality of their children who in the future are also likely to be at risk for DR-NCDs based on lifestyle and food choices patterns [11]. The long-range effects on social disparities in health over historical and epidemiological time is therefore a potential hazard, and hence the need to ensure that policies to combat such disparities are contextually specific [29]. This case study suggests socioeconomic differences across subpopulations should be considered when addressing nutrition policy questions.

## Conclusion and Recommendations

The limitation of this study is sample size and a focus on a community of urban adult women. However given this community approach, the case study presented here identifies ethnicity and income as socioeconomic factors contributing to a less satisfactory diet quality for adult women living in an urban community in Malaysia. Policy interventions that are community-based may be required to facilitate budget constrained families to improve the quality of their diets given rising inflation. Multiethnic populations in Asia are common to Malaysia, Singapore, Indonesia and Brunei. This case study has implications for nutrition educators in these countries tacking multiracial populations.
